# Daydreaming optometry and computer vision syndrome - the necessity of a Social Marketing campaign in Romania

**Published:** 2020

**Authors:** Mădălina Gheorghe Consuela

**Affiliations:** * Philologist, Authorized translator

Computer Vision Syndrome (CVS) has become a real problem during the past couple of years, as it appears in individuals due to prolonged computer, cell phone and tablet use. In addition, different associations, such as the Ontario Association of Optometrists (OAO) has recommended regular breaks, when looking at digital screens. It is already known that prolonged computer, cell phone and tablet usage may cause dry eyes, neck and shoulder pain, headaches, etc. All these eye problems may have as outcomes itchy eyes, blurred eyes and many other eye problems.

In order to prevent these eye problems, a rule has been established by OAO, and that is, to look away from the screen every 20 minutes, for 20 seconds, and focus on something that is located 20 feet away. Consequently, optometrists advice adult individuals to close their eyes and daydream for 20 seconds. At the same time, children are recommended to look away and daydream on occasion, because, by doing so, they may prevent CVS, which includes affections, such as eye strain and burning eyes. 

The following tips are offered to prevent and reduce eye problems of kids: the height and setting of the computer should be checked; the glare on the computer screen should be checked, meaning that windows or other light sources should not be visible in the monitor; the amount of lighting in the room should be reduced to match the computer screen; continuous blinking is recommended.

Provided that taking short 20 seconds breaks at every 20 minutes is almost impossible during a regular eight-hour work day, when most of the people spend their entire time staring into a screen, a Social Marketing campaign should also be implemented in Romania, with the aim to raise the awareness regarding this emergent modern “disease” by bonding, bridging and linking the social issues and behavior change, and ensuring, in fact, a balance. 

Most of the times, Social Marketing campaigns are elaborated to assess a behavior change in a population, but are not going to be successful if certain strategies are not implemented. For instance, the determinants of a successful adopted health behavior depend on immediate environment conditions that encompass peers, local community, significant others and family, on wider social context elements such as societal norms, cultural symbolism, structural issues and social and economic conditions, and, in addition, on personal characteristics, with a specific interest in individual particularities, as goals, aspirations, self-efficacy, education, symbolic needs and skills.

**Fig. 1:**
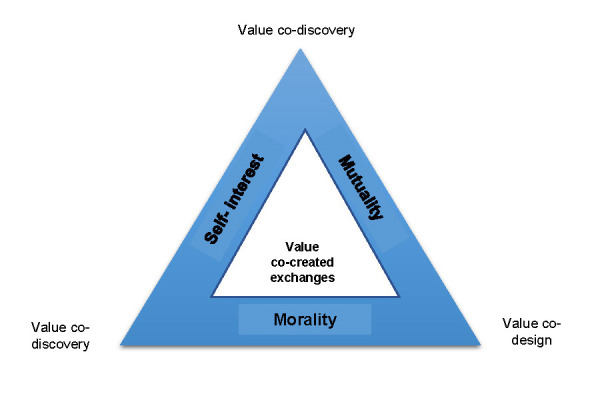
Social Marketing’s value triad Source: Hastings G, Domegan C (Editors). Social Marketing. Rebels with a cause. 2017, Routledge, 25

In conclusion, I strongly believe that the efficient application of Social Marketing principles takes the shape of the Social Marketing’s Value Triad, as illustrated in **Fig. 1**. 

In Ophthalmology, it is acknowledged the fact that value is expressed subjectively and “is in the eye of the beholder”. Basically, it is important to refer to values because they are beliefs, ideas, experiences, emotions, and motivate actions and transcend to specific actions and situations. 

**Assist. Prof. Gheorghe Consuela-Mădălina, PhD,** **Philologist, Authorized translator**

